# Ultrasonic Al_2_O_3_ Ceramic Thermometry in High-Temperature Oxidation Environment

**DOI:** 10.3390/s16111905

**Published:** 2016-11-11

**Authors:** Yanlong Wei, Yubin Gao, Zhaoqian Xiao, Gao Wang, Miao Tian, Haijian Liang

**Affiliations:** 1 Science and Technology on Electronic Test & Measurement Laboratory, North University of China, Taiyuan 030051, China; ybgao@nuc.edu.cn (Y.G.); wanggao@nuc.edu.cn (G.W.); 18835101671@163.com (M.T.); 18334789051@163.com (H.L.); 2 North Automatic Control Technique Research Institute, Taiyuan 030006, China; michaelxzq@Outlook.com

**Keywords:** ultrasonic thermometer, high-temperature oxidation, delay time, ultrasonic wavelets

## Abstract

In this study, an ultrasonic temperature measurement system was designed with Al_2_O_3_ high-temperature ceramic as an acoustic waveguide sensor and preliminarily tested in a high-temperature oxidation environment. The test results indicated that the system can indeed work stably in high-temperature environments. The relationship between the temperature and delay time of 26 °C–1600 °C ceramic materials was also determined in order to fully elucidate the high-temperature oxidation of the proposed waveguide sensor and to lay a foundation for the further application of this system in temperatures as high as 2000 °C.

## 1. Introduction

High-temperature measurement is critically necessary in many fields including aerospace, production, energy, metallurgy, and various sectors of the chemical industry. It is crucial for the development of advanced engines and hypersonic missiles, for example, and other pieces of equipment subjected to ultra-high temperature environments due to combustion or high-speed friction. The in situ dynamic acquisition of temperature parameters in an ultra-high temperature environment (>1500 °C) for a long period of time (>0.5 h) is the universal test for any technology subject to military requirements.

Existing approaches to high-temperature measurement include the traditional thermocouple, thermal resistance, and other contact-type and non-contact type measurement instruments. For example, thermocouple temperature measurement range types K and N do not exceed 1100 °C; the platinum-rhodium thermocouple (R, S, and B) can be used intermittently (for several hours) up to 1800 °C and continuously (for several hundred hours) at temperatures up to about 1750 °C [1]. Tungsten-rhenium thermocouples (W-3Re/W-25Re) and (W-5Re/W-26Re) cannot function in oxidative environments [2]. Non-contact instruments work by measuring the thermal radiation of a detection target and obtaining the temperature parameters, but they do not work perfectly. Any non-contact temperature measurement process is affected by both the surrounding environment and the emissivity of the material object being tested. To accurately measure the temperature of different objects, it is necessary to first calibrate material emissivity through a high-precision device to ensure that the temperature measurement precision is lower than that of the sensor.

Ultrasonic technology has been instrumental in developing new approaches to temperature measurement in ultra-high temperature environments [3,4]. In 2002, the Korea Atomic Energy Research Institute (Kil-MO Koo) used ultrasonic thermometry to measure core temperature in LAVA (Lower-Plenum Arrested Vessel Attack) at 2200 °C [5]. In 2010, the Idaho National Laboratory tested an improved, ultrasonic in-pile sensor for temperature detection during irradiation testing [6,7]. Experiments showed that UO_2_ melting with an ultrasonic sensor embedded in a molten pool has a temperature near 3133 K, which is well in accordance with the expected value of 3123 K [8,9]. Though there have been many valuable contributions to the literature, there have been no studies to date on the use of ultrasonic temperature measurement techniques in high-temperature oxidation environments. In this study, we designed a temperature measurement scheme with Al_2_O_3_ anti-oxidation ceramic as an ultrasonic temperature measurement sensor, laser-carved sensitive segments of the measurement device, and implemented static calibration on the sensor. The device yielded accurate calibration curves, as discussed below.

## 2. Principle

Ultrasonic thermometry, which has been developed extensively within the past fifty years, is based on the theoretical principle that there are certain functional relationships between the propagation velocity of ultrasonic waves in gas, liquid, and solid at certain temperatures [10,11,12]. Ultrasonic guided-wave temperature measurement enables the user to measure medium temperature according to the sound velocity of ultrasonic waves in the particular medium [13,14,15].

The velocity of a longitudinal ultrasonic wave propagating in a thin rod can be expressed as follows:

(1) $$V(T)=\sqrt{\frac{E(T)}{\rho (T)}}$$

where *E* denotes the Young’s modulus of the waveguide material, *ρ* is density, and *V*(*T*) is the velocity of a longitudinal ultrasonic wave in the bar.

**Figure 1:**
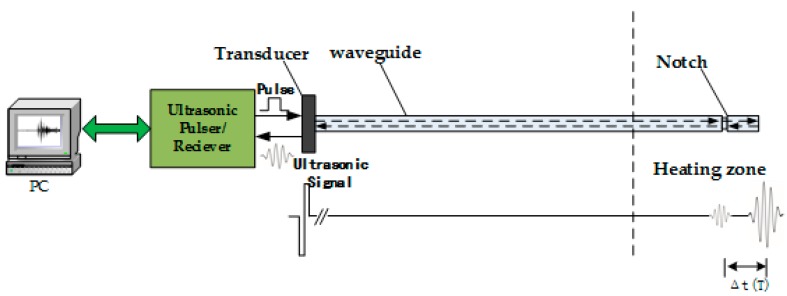
Structure diagram of temperature measurement system established via ultrasonic pulse method.

Because it is difficult to determine the precise form of Equation (1) in theory, the relationship between ultrasonic velocity and temperature is usually determined through experimentation. In our experiment, the changes in velocity and temperature were measured using the device shown in Figure 1. A computer controls the ultrasonic pulser/receiver to stimulate a narrowband pulse, which turns into an ultrasonic pulse signal propagating in the waveguide rod through a piezoelectric ultrasonic transducer. Ultrasonic pulse signals are transmitted along the waveguide rod, then a signal reflection emerges in the notch, the waveguide end, and other locations; ultrasonic echo signals are converted to electrical signals through the piezoelectric transducer, then the ultrasonic pulser/receiver amplifies the electrical signals and transmits them to the signal acquisition device. The average temperature corresponding to the sensor segment, i.e., the delay time difference of echoed signals at the notch and signals at the waveguide end, is represented by Equation (2):

(2) $$\Delta t(T)=\frac{2\Delta l}{V(T)}$$

where Δ*t* denotes the delay time, Δ*l* is the length of the sensitive segment, and *V*(*T*) is the average velocity of longitudinal wave in sensitive segment. If there were several temperature-sensitive sensor segments, could be used to monitor the gradient temperature distribution of the detected object.

## 3. Ultrasonic Thermometry Requirement and Development

### 3.1. Sensor Material

**Figure 2:**
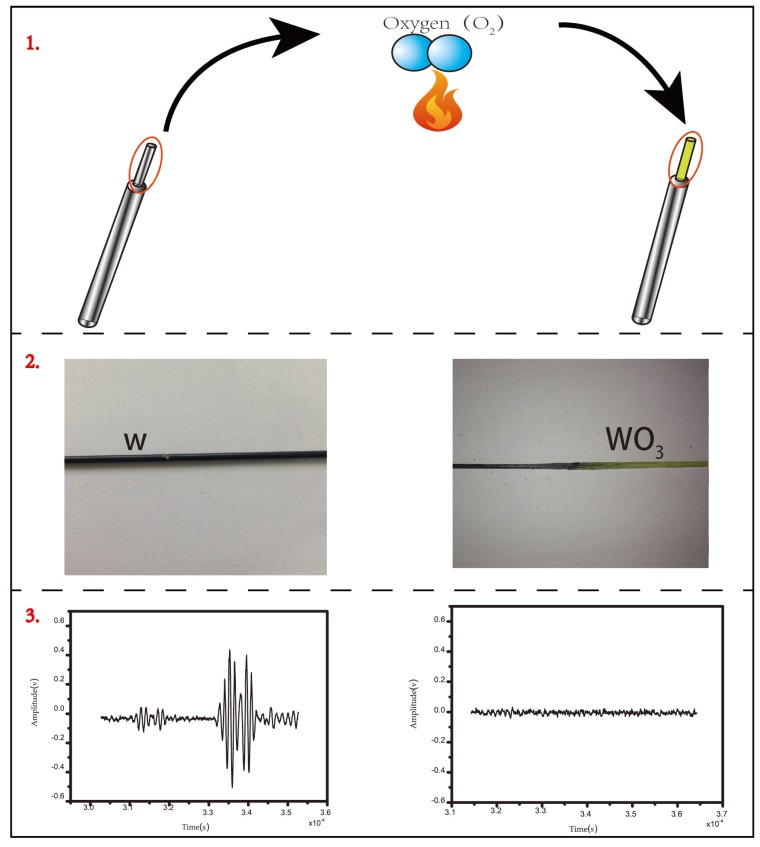
Schematic diagram of tungsten oxide generation.

Temperature measurement sensor materials have high melting points (2000 °C–3000 °C) and are stable under high temperatures. Most previous studies have considered refractory metals (metals with a melting temperature above 3000 °C such as tungsten, rhenium, tungsten-rhenium alloy, and tungsten-thorium alloy) as ideal candidates for sensor materials. Such metals have many advantages, including high melting point, small acoustic impedance, marked change in velocity with temperature change, and convenient operation [16]. For the temperature testing of engine combustion chambers and fully-burnt fossil fuels, when refractory metals are selected as the sensitive elements of ultrasonic temperature measurement sensors, the melting point can cover the temperature of the testing environment but the material tends to be oxidized. Oxidation damages the temperature-sensitive interval degeneration and changes the Young’s modulus and density of the material, thus making the measured data deviate from the true value. At 900 °C, tungsten reacts with oxygen, transforms into tungsten trioxide and oxides, and the Young’s modulus and density change considerably; the ultrasonic signals disappear in the notch and the experiment fails. As shown Figure 2, tungsten reacts when heated in a high temperature oxidization environment and generates tungsten oxide.

In this study, we adopted an anti-oxidation ceramic as the ultrasonic sensor. Because they are sintered at ultra-high temperature, industrial ceramic materials can function for a long time at temperatures above 1800 °C [17]. The melting points of Al_2_O_3_, MgO, ZrO_2_, and HfO_2_, as shown in Table 1, are all above 2000 °C. We utilized Al_2_O_3_ ceramic (99.95% purity), which has several notable advantages over similar ceramics such as high temperature resistance, corrosion resistance, wear resistance, small acoustic impedance, and stable chemical properties in high temperature oxidation environments.

### 3.2. Sensor Segment Design and Fabrication

**Figure 3:**
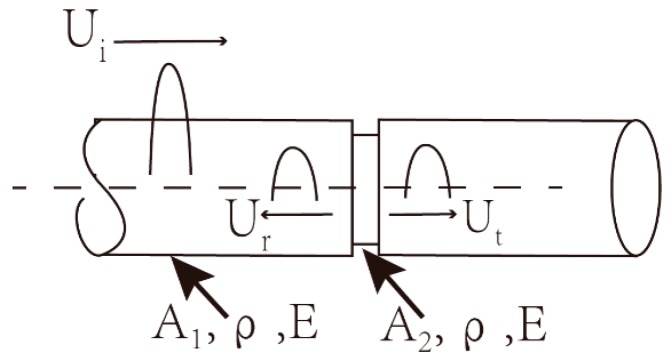
Incident, reflected, and transmitted waves at waveguide junction.

The diameter, notch depth, notch shape, and length of the temperature sensitive segment were our primary sensor design factors. Propagating ultrasound in the solid is accompanied by dispersion, which broadens the wave envelope and affects the ultrasonic temperature measurement precision [18,19,20]. To prevent the dispersion of ultrasonic propagation in the rod, the diameter of the waveguide rod should be lower than 1/10 of the longitudinal wavelength [4]. For an ultrasonic transducer at 2.5 MHz, this means that the diameter must be lower than 1 mm. In our sample, we carved the annulus notch via laser at the place of the waveguide rod 25 mm away from sensor end; the sound waves at the notch produced reflection signals accordingly. In general, reflection signals are generated mainly due to acoustic impedance mismatch at the notch of the acoustic waveguide, which results in reflection and transmission of the ultrasonic wave as shown in Figure 3.

Acoustic impedance can be calculated as follows [7]:

(3) $$Z=\frac{\rho c}{A}$$

where *ρ* denotes material density, *c* indicates the velocity of sound, and *A* is the cross-sectional area of the acoustic waveguide. Based on ultrasonic impedance equation, we can deduce the ultrasonic reflection coefficient as follows [21,22]:

(4) $$R=\frac{Z_{2}-Z_{1}}{Z_{2}+Z_{1}}$$

After plugging Equation (3) into Equation (4), in which *Z*_1_ is the impedance of cross-sectional area *A*_1_, *Z*_2_ is the impedance of the cross-sectional area *A*_2_, and *R* is the reflection coefficient, we obtained the following:

(5) $$R=\frac{d_{1}^{2}-d_{2}^{2}}{d_{2}^{2}+d_{1}^{2}}$$

The ultrasonic reflection coefficient also corresponds to the energy of reflected and initial signals:

(6) $$R=\frac{E(U_{r})}{E(U_{i})}=\frac{A_{r}^{2}}{A_{I}^{2}}$$

where *E*(*U_r_*), *E*(*U_i_*) denote the reflected wave energy and initial wave energy, *A_r_* is the amplitude of the reflected wave, and *A_I_* is the amplitude of the initial wave.

After plugging Equations (5) and (6) into Equation (4), we obtained the following relationship between the cross-section diameter and signal amplitude:

(7) $$\frac{A_{r}^{2}}{A_{I}^{2}}=\frac{Z_{2}-Z_{1}}{Z_{2}+Z_{1}}=\frac{d_{1}^{2}-d_{2}^{2}}{d_{2}^{2}+d_{1}^{2}}$$

where *Z*_1_ is the impedance of the cross-sectional area *A*_1_, *Z*_2_ is the impedance of cross-sectional area *A*_2_, and the diameters of cross-sectional areas *A*_1_ and *A*_2_ are *d*_1_ and *d*_2_, respectively.

**Figure 4:**
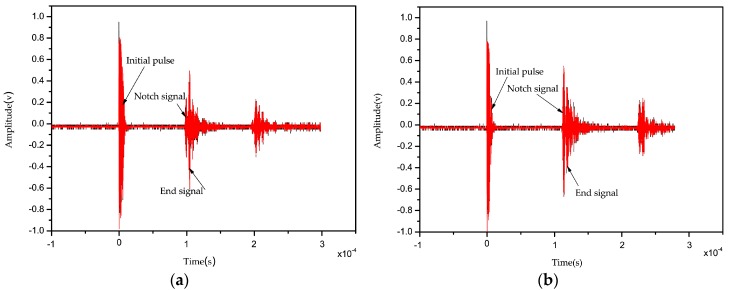
Notch depth with (**a**) 0.1 mm; (**b**) 0.3 mm notch signal amplitude.

According to Equation (7), the intensity of the reflection signal is related to notch depth. We carved the notch in our sample via laser engraving, as mentioned above. The laser power was controlled by adjusting the size of the input current pulse and pulse width, which ultimately changed the amplitude of the reflected signal at the notch. Figure 4 below shows the signal amplitude at notches with depths of 0.1 mm and 0.3 mm; notch depth is smaller when the reflection coefficient and notch signal are smaller.

### 3.3. Ultrasonic Equipment

The sensitive segment of the sensor is only 25 mm and the delay time between the reflected signal of the notch and the end at room-temperature is 5~6 μs. The width of a sound wave must be below 3 μs to ensure sufficient time difference between the two signals. The transducer excitation was run at a frequency of 2.5 MHz to distinguish between the signals.

The ultrasonic temperature measurement system includes a CO-7 pulsed ultrasonic instrument with an SIUI model (Shantou Institute of Ultrasonic Instruments, Shantou, China) 100 MHz data acquisition (DA) card, an Al_2_O_3_ acoustic waveguide sensor with a length of 600 mm and diameter of 1 mm, and a 2.5 MHz ultrasonic transducer. The ultrasonic pulse meter can stimulate the potential pulse with an amplitude of 600 V, pulse width of 2 μs, and repetition frequency of 50–500 Hz. A potential pulse can stimulate a 2~3 μs ultrasonic signal in an ultrasonic transducer where the signal produces a reflected signal at the sensor notch and end. These signals are amplified after a series of linear amplifications and ultimately stored in the DA. According to the calibration curve of the tungsten-based ultrasonic temperature measurement sensor, the rate of change in delay time with changes in temperature is 0.9 ns/K/cm [23], so the delay time change is 90 ns when the temperature of the environment rises by 100 °C. To effectively calculate the delay time at different temperatures, it is necessary to restore the characteristics of the notch and end signal effectively—i.e., it is necessary to apply high frequency acquisition cards, so we used a 100 MHZ acquisition card for this purpose.

## 4. Laboratory Experiment and Test Result

**Figure 5:**
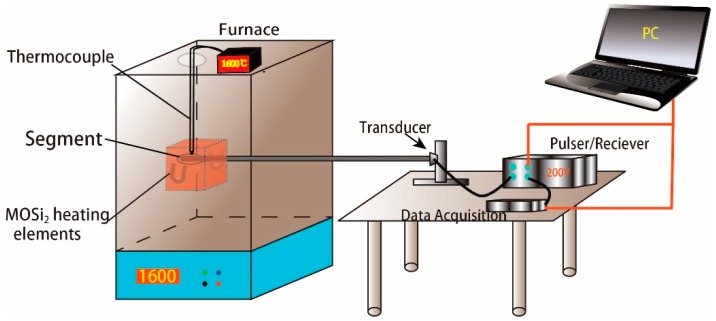
Schematic diagram of the test setup.

**Figure 6:**
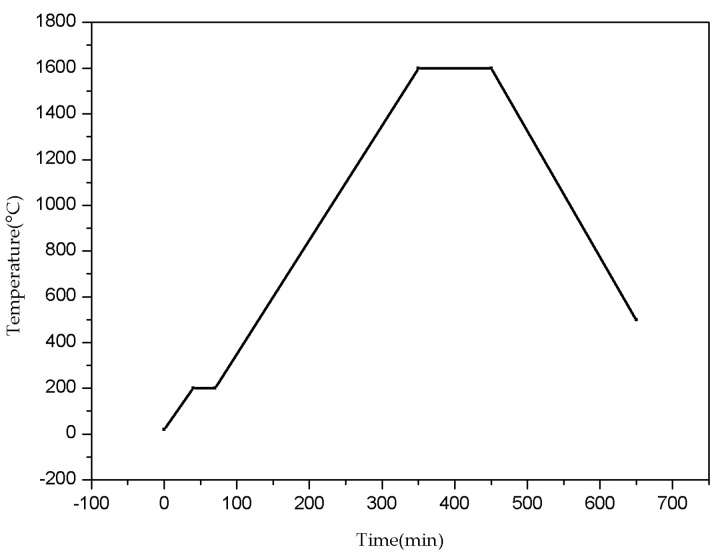
Muffle furnace temperature curve.

As shown in Figure 5, we used a muffle furnace was used for calibration that contains MOSi_2_ heating elements suited to temperatures up to 1650 °C, (or 1600 °C for long-term use). Figure 6 shows the temperature curve of this furnace. The structure of the heating zone is a 200 mm × 250 mm × 200 mm chamber. According to the temperature gradient experiment, the temperature gradient between any two points in the chamber is about 6 °C. To decrease calibration error, we added another type-B thermocouple with precision of 0.3% beside the segment for the static calibration of temperature from 26 to 1600 °C. A carefully assembled temperature sensor was placed into the heating zone, then an ultrasonic pulse sending and receiving apparatus was connected to the ultrasonic transducer, DA, and PC. The ultrasonic pulse sending and receiving apparatus stimulated the transducer through high voltage and to create ultrasound; the ultrasonic signal was amplified through the pulse sending and receiving apparatus and stored in the PC through the acquisition card.

After denoising and filter-processing the obtained data [24,25,26,27], the delay time between the notches and end signals was calculated via the cross-correlation algorithm. The delay time values as-calculated are described in the figures below.

## 5. Discussion

**Figure 7:**
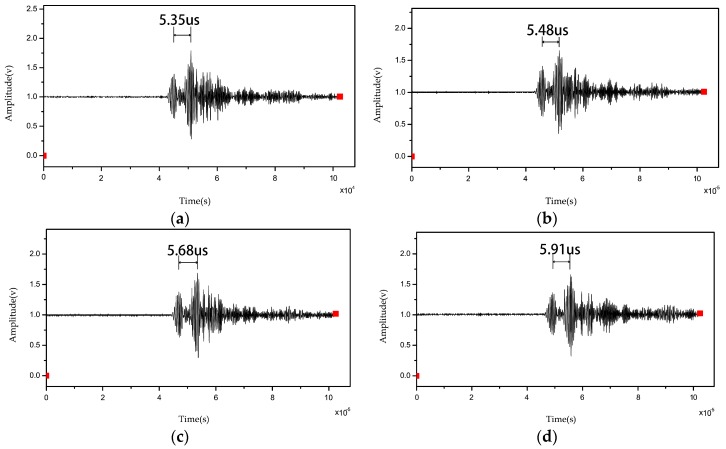
Delay time at (**a**) 26 °C; (**b**) 500 °C; (**c**) 1000 °C; (**d**) 1500 °C.

**Figure 8:**
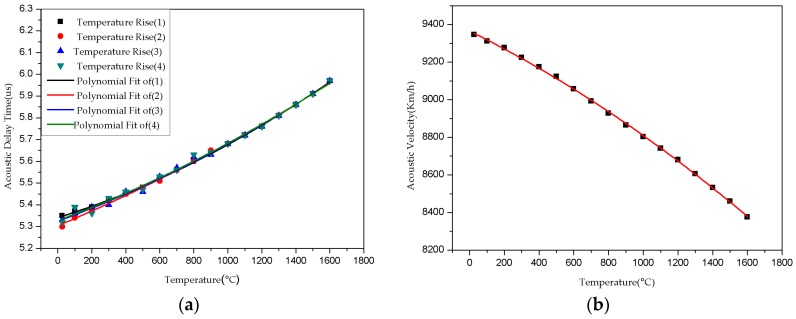
Curve for (**a**) Al_2_O_3_ ultrasonic thermometer sensor calibration; (**b**) Al_2_O_3_ velocity with temperature.

According to the experiment described above, in which an Al_2_O_3_ temperature measurement sensor was used as an acoustic waveguide, there is no difference in signal amplitude between high-temperature and room-temperature. In effect, then, Al_2_O_3_ ceramic don’t react with oxygen under high-temperature environment. Figure 7 shows that delay time increases gradually as temperature increases across 26 °C, 500 °C, 1000 °C, and 1500 °C points. The Al_2_O_3_ ultrasonic thermometer sensor calibration curve indicates linear delay time values with temperature (Figure 8a). The signal amplitude attenuation of ultrasonic signals collected at 1500 °C is smaller compared to that at room-temperature. These observations altogether suggest that the proposed ultrasonic sensor can be employed at temperatures as high as 1800 °C.

According to the Al_2_O_3_ ultrasonic thermometer sensor calibration curve, the delay time information directly indicates the corresponding temperatures. The calibration curve is slight non- coincident at low temperature, however, above 1000 °C the curve is highly repeatable. We can measure delay time to obtain the corresponding temperature. According to Equation (2), it is possible to determine the Al_2_O_3_ ceramic acoustic velocity as a function of temperature (Figure 8b).

**Figure 9:**
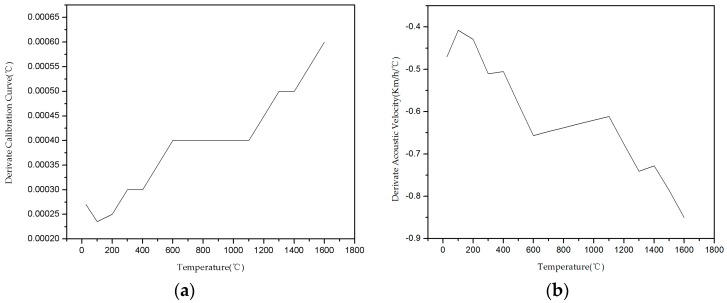
Derivate curves: (**a**) Calibration curve; (**b**) Acoustic velocity curve.

We defined the calibration curve derivative as the sensor sensitivity. The derivative can be obtained via Equation (8), where the first term represents the dilation contribution and the second term represents the effect of speed of acoustic variations shown in Figure 9a was calculated according to the point (*T*, *t*) and the previous and subsequent points of two average slopes (the only exception to this was the last point, which was calculated according to only itself and the slope of the previous point.)

(8) $$\frac{\partial \Delta t}{\partial T}=\frac{2}{V(T)}*\frac{\partial l}{\partial T}-\frac{2l}{V^{2}(T)}\frac{\partial V(T)}{\partial T}$$

where *V*(*T*) denotes acoustic velocity with temperature, *T* is the temperature, *l* is the length of the segment, Δ*t* is the delay time.

**Figure 10:**
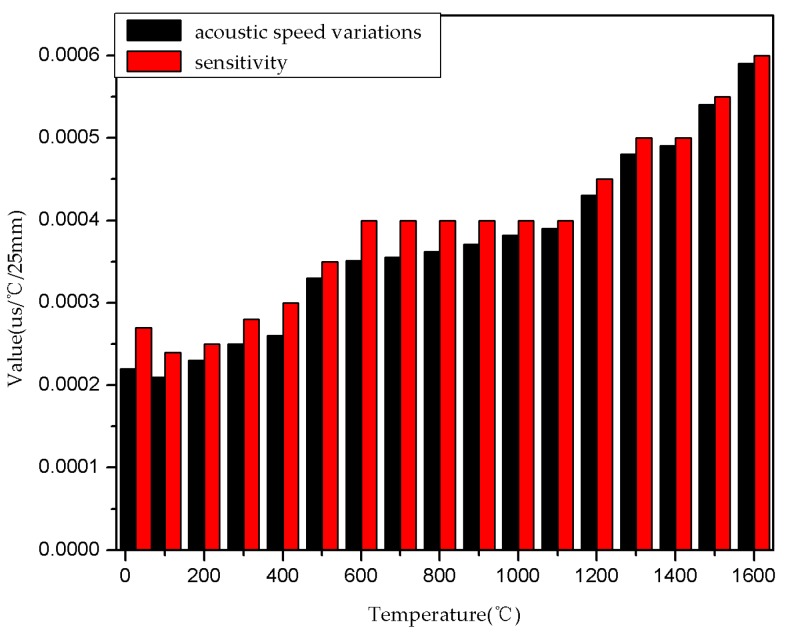
Value of sensitivity and acoustic speed variations.

As the same method, we can get the acoustic velocity derivative, and as the curve in Figure 9b shows, the differential coefficient of Al_2_O_3_ ceramic acoustic velocity with respect to temperature is less than zero. The speed of acoustic variations and the sensor sensitivity increase as temperature increases, which makes the calibration curve highly repeatable. As shown in Figure 7, the calibration curve is more coincident above 1000 °C because the sensitivity of delay time to temperature is about 0.2~0.3 ns/°C/cm (for 25 mm-long measurement zones). From Equation (8) and Figure 9, the value of sensor sensitivity and the acoustic speed variations are calculated, and illustrated in Figure 10. It is shown that the effect of acoustic speed variations is more important than dilation for sensitivity.

Calibration curve errors have three main causes:

- The thermocouple precision is ±3 °C in its calibration range;
- The temperature gradient between any two points in the furnace is 6 °C;
- The transmission line distortion and receiver electronics instabilities are within ±10 ns (±30 °C).

## 6. Conclusions

Based on the basic principles of ultrasonic temperature measurement, this paper examined several ultrasonic high temperature measurement methods. Ultrasonic measurement technology is commonly used to evaluate nuclear reactor core temperatures or to measure the temperature of other objects with temperature gradients. Ultrasound temperature measurement technology is used in anaerobic environments, or a protection sheath is equipped with an outside sensor to prevent oxidation; this greatly limits the application of ultrasonic measurement in aerobic, high-temperature environments.

We designed an ultrasonic temperature measurement system with Al_2_O_3_ as the acoustic waveguide material that can be used in an oxidative environment. The system can work effectively in oxidation environments even without a protection sheath, and thus has a wide application range. We ran several experiments to demonstrate that the system can work stably under 1600 °C. We obtained 26–1600 °C ultrasonic signals, calculated the time differences between the notch and end signals through a cross-correlation algorithm, and calculated the delay time under different temperature points while effectively drawing the 26–1600 °C temperature-delay time change curve.

The upper temperature limit of our experimental furnace is 1600 °C, so we were only able to measure the relationship between the 1600 °C of temperature and velocity here though ultrasonic signal attenuation is relatively small at 1600 °C. In the future, we plan to explore the relationship between temperatures below 2000 °C and velocity with Al_2_O_3_ as the sensitive element of temperature measurement. We also plan to explore this system’s applicability in temperatures above 2000 °C by replacing the acoustic waveguide materials with ZrO_2_ and MgO.

## Figures and Tables

**Table 1:** Anti-oxidation ceramic with melting point.

Material	Melting Point
99% Al_2_O_3_	2050 °C
MgO	2800 °C
ZrO_2_	2700 °C
HfO_2_	2850 °C
